# A Validation Study of the COPSOQ III Greek Questionnaire for Assessing Psychosocial Factors in the Workplace

**DOI:** 10.3390/healthcare13161980

**Published:** 2025-08-12

**Authors:** Aristomenis Kotsakis, Demetris Avraam, Maria Malliarou, Elpidoforos S. Soteriades, Constantinos Halkiopoulos, Michael Galanakis, Michael Sfakianakis

**Affiliations:** 1 Department of Management Science and Technology, University of Patras, 26334 Patras, Greece or a.kotsakis@unipi.gr (A.K.); halkion@upatras.gr (C.H.); 2 Department of Business Administration, University of Piraeus, 18534 Piraeus, Greece; msfakian@unipi.gr; 3 Department of Public Health, University of Copenhagen, 1014 Copenhagen, Denmark; demetris.avraam@sund.ku.dk; 4 Laboratory of Education and Research of Trauma Care and Patient Safety, Department of Nursing, University of Thessaly, 41500 Larissa, Greece; 5 Healthcare Management Program, School of Economics and Management, Open University of Cyprus, 2020 Nicosia, Cyprus; elpidoforos.soteriades@ouc.ac.cy; 6 Hellenic Open University, 26335 Patras, Greece; mgalanakis@ekdd.gr; 7 National Centre for Public Administration and Governance—EKDDA, 17778 Athens, Greece

**Keywords:** COPSOQ, psychosocial stress, risk assessment, work factors, Greek validation study, organizational diagnosis, psychosocial risk management, psychosocial work environment, work environment, emerging risks, psychosocial risk analytics, management interventions, human-risk monitoring, psychosocial well-being

## Abstract

Background: Over the past two decades, the Copenhagen Psychosocial Questionnaire (COPSOQ) has been established as a valid instrument to measure psychosocial stress at work. Currently, the COPSOQ international network is responsible for monitoring and improving the COPSOQ. In 2019, a new questionnaire was published, and the Greek version is now being validated. The aim of the current study was to assess the reliability and validity of the psychometric properties of the Greek long version of the Copenhagen Psychosocial Questionnaire III (COPSOQ-III-GR). Methods: The measurement qualities of the Greek COPSOQ III have been explored in accordance with the usual requirements of a validation study, as defined by DIN EN ISO 10075-3. A sample of observations from 2189 participants surveyed with the COPSOQ in Greece was used to validate the current version with appropriate statistical analyses. Exploratory factor analysis was used to assess the statistical relationships for many scales. Results: With its 108 items and 40 scales, the Greek COPSOQ III includes all internationally validated psychosocial workplace factors that remain comparable (~72%) with the COPSOQ III German version content. In addition to the primary results, congruence with widely used theoretical approaches such as the demand–control (−support) model (DCM) or the job demands–resources model (JDR) is generally satisfactory. In summary, our validation study for the Greek COPSOQ III version showed adequate reliability and validity, which is in line with the findings of the COPSOQ III questionnaire from other European countries, and it is also compatible with the validation of the German COPSOQ III. Our regression analysis revealed that 34 psychosocial workplace factors (34 “context” scales) could adequately predict the scores of the satisfactory and health scales (6 “outcome” scales). The analysis also revealed the top five predictors (context variables) for each of the six “effect” scales (outcome variables). Conclusions: With the launch of COPSOQ III in Greece, current and new workplace psychosocial aspects could be explored, since COPSOQ III (GR) appears to be a valid and reliable instrument for enterprise research and risk assessment.

## 1. Introduction

According to the European Organization for Health and Safety at Work, psychosocial risks and occupational stress are among the greatest challenges in the fields of occupational safety and health. They significantly affect the health of individuals, businesses, and national economies. Psychosocial stressors threaten employees and managers within organizations in the public and private sectors [1,2]. In the past 12 years of the economic crisis in Greece, an “exponential” escalation of these psychosocial hazards has been experienced, which—to a greater extent—has worsened during the COVID-19 era and post-COVID-19 era at both the national and international levels [3,4,5,6].

Psychosocial risks arise from inadequate planning, organization, and management of work, as well as from an unhealthy social context of the workplace environment, and may lead to negative physical, psychological, and social outcomes for employees, such as work stress, burnout and/or depression [7,8,9]. Some examples of working conditions that may lead to psychosocial risks include excessive workload, conflicting demands and ambiguities regarding employees’ roles, a lack of participation in decision-making, a lack of influence on how work is carried out, poor management of organizational change, job insecurity, ineffective communication, a lack of support from management or colleagues, psychological and sexual harassment, violence, etc. [10,11,12].

A psychosocially healthy work environment increases performance and personal development and reinforces the physical and mental well-being of employees [13,14]. Employees experience stress attacks when the demands of their work are excessive and exceed their ability to cope with them. In addition to mental health problems, workers suffer from prolonged stress with a high risk of developing serious physical health problems, such as cardiovascular disease or musculoskeletal problems [15,16]. At the organizational level, negative consequences may include poor overall business performance, increased absenteeism, truancy (instances of employees showing up to work while ill and unable to function effectively), and increased accident rates and injuries. Stress-related absences tend to be longer than absences related to other causes, and work-related stress contributes to increased early retirement rates. The costs to businesses and society are estimated to be significant and amount to billions of euros [17,18].

Furthermore, leadership is another crucial psychosocial aspect that plays a significant role in managerial interventions and policies. The impact of leadership behaviour and style on employees’ health and well-being has been identified as a noteworthy psychosocial risk factor [19,20]. Effective leadership includes actively engaging health professionals by attentively listening to their problems and expectations, facilitating collaborative team planning, and appropriately allocating workloads. By carefully considering employees’ psychological needs and implementing supportive leadership practices, managers and policymakers can cultivate a work climate that fosters well-being and productivity [21,22,23]. Workplace bullying is an additional psychosocial aspect that impacts management responses and legislation. Research has indicated that several psychosocial characteristics within the workplace, including but not limited to quantitative demands, job control, role expectations, leadership conduct, and the social climate, are associated with workplace bullying. The primary emphasis of interventions to prevent workplace bullying should be enhancing psychosocial working conditions, decision-making processes, leadership abilities, and other organizational aspects [24,25,26]. By considering these various elements, managers and policymakers can establish a work atmosphere that fosters respect and inclusivity, enhances employee well-being, and mitigates the likelihood of bullying [27,28,29].

In Greece, employers have a legislative obligation to assess psychosocial risks, such as the risks of violence and harassment, including sexual harassment, and are expected to take measures to prevent and control such hazards in the workplace (amendment of paragraph 6 of article 42 of Law 3850/2010) [30]. Employers are required to take specific measures to prevent and address violence and harassment at work; demonstrate zero tolerance for such incidents or behaviour when receiving and being called upon to manage related complaints; and provide information and training on the risks, prevention, protection and obligations of those involved in accessible formats (Law 4808/2021) [31].

The Copenhagen Psychosocial Questionnaire (COPSOQ) was originally developed in 2000 for research purposes at workplaces in Denmark, and it has since been validated in 18 countries [32]. It covers all important psychosocial factors at work, considering the leading concepts and theories of occupational health and well-being [33,34]. The COPSOQ International Network was founded in 2009 to promote scientific research and workplace risk assessment via the COPSOQ tool. The COPSOQ may be used by governments, universities and research institutions, enterprises, and social agents from Europe and other countries worldwide [35]. The network emphasizes the importance of validation studies supporting tailored national versions of the instrument.

The COPSOQ is a comprehensive questionnaire that covers a wide variety of dimensions, describes psychosocial working conditions, and is considered an instrument for research and psychosocial risk prevention in the workplace [35]. Researchers [33] have reported that the COPSOQ can be used as a valid and reliable tool for psychosocial risk assessment in the workplace, which is why it has been used in thousands of enterprise-based risk assessment applications [36]. It can capture a broad range of psychosocial dimensions [37] and is part of a systematic occupational safety and health management system [38].

The questionnaire covers a broad range of aspects of currently leading concepts and theories, such as the job characteristics model, the Michigan organizational stress model, the demand–control (support) model, the sociotechnical approach, the action–theoretical approach, the effort–reward–imbalance model, and the vitamin model [37]. COPSOQ I and II have short, middle, and long versions [38]. A new version of the questionnaire (COPSOQ III) was developed by the International COPSOQ Network as an update of the previous two versions [37,38]. A set of core items of the COPSOQ III is strongly recommended to be included in the national short, middle, and long versions of the questionnaire [32]. Many items and scales of COPSOQ III have been included in the COPSOQ questionnaire since 2005. In total, 58 of the 84 items (approximately 70%) are identical [39].

The new version of COPSOQ III is designed to allow flexible adaptation to national and industry-specific contexts without compromising the potential for international comparisons and comparisons over time. National versions can be established by the national COPSOQ teams of each country on the basis of all “core” items included in the original source of the COPSOQ questionnaire [39] and supplemented with additional items labelled “middle” or “long” to form a reliable and relevant tool in the given context. Therefore, all future national versions include the same mandatory core items, while the total number of items in the scales and the number of scales are allowed relative flexibility [32,40].

COPSOQ III has been validated for the German, Spanish, French, Swedish, and Dutch languages [41,42], as well as in many countries worldwide (e.g., Canada and Chile). There is also an important validation study (available also on the COPSOQ-Network website) with the content of a joint-effort validation for the international middle version of COPSOQ III. An international validation study could be used as a linguistic “common point of reference” for both scientific research and practical implication-related projects [32].

The Greek validation study project was launched in October 2017 at the University of Piraeus with the aim of adapting and validating key international tools for use in the Greek context. The study explored various aspects of employability and professional preparedness among students and graduates. Preliminary findings indicated a strong relevance between academic training and employability competencies, along with the need for enhanced practical learning opportunities. Some initial results were presented at the Employability for the 21st Century International Conference in Leuven, Belgium (September 2018) and are discussed in more detail in Dr Kotsakis’s doctoral thesis [43,44].

The aim of the current study was to assess the reliability and validity of the psychometric properties of the Greek long version of the Copenhagen Psychosocial Questionnaire III (COPSOQ-III-GR).

## 2. Materials and Methods

The study was conducted in Greece in the context of both doctoral and postdoctoral research activities at two Greek universities (the University of Piraeus-Greece and the University of Patras-Greece) between 2017 and 2022. Owing to data quantity and quality-related restrictions and our overall filtering process results, we ultimately included only the data from the University of Piraeus research activities. The participants were employees from different public and private organizations. In both pre-pandemic and post-pandemic periods of our survey we selected the convenience sampling method, which is a non-probability sampling method, because it was the easiest for our researcher for an effective rollout of the survey, due to geographical proximity, availability at a given time, and willingness to participate in our research. We chose the above method of non-probability sampling because our population parameters were not possible to individually identify for both pre-pandemic and post-pandemic periods. Also, due to GDPR restrictions and compliance, our cloud-app did not require users (participants) to create a registration account as a mandatory step to fill in the COPSOQ questionnaire form. So, our sampling method was at higher risk for possible research biases in comparison with probability sampling, and particularly for possible sampling bias. To collect our data, we published via the cloud-platform of the academic radar app. In addition, we forwarded the link to 172 academic and business colleagues in Greece (academic teachers, business consultants, HR managers, and HR generalists), asking them whether they wanted to participate in our research. The overall (average) response rate was 22.5% within a five-year period. We continued to ask for additional participation and relevant support until the desired sample size (more than 2000 valid responses) was reached.

### 2.1. Ethics Statement

The survey was implemented in accordance with the COPSOQ International Network Research Guidelines and in compliance with the General Data Protection Regulation (GDPR) of Greece. The research study, which complied with the COPSOQ Network Research Guidelines with respect to anonymity, confidentiality, and research ethics and the overall research study protocol, was approved by the Research Ethics Committee of the University of Piraeus. The respondents were given personalized credentials to access the questionnaire through a provided URL to the Academic Research Radar. A written user manual was given to the respondents with instructions on how to access the survey and a notice that by clicking on the link to proceed to the survey, they were providing consent to participate. Participation was performed anonymously, and the system was not able to identify an individual by their name or any other direct identifier. The system initiating the survey was used as a record of consent.

### 2.2. Sample

The aim of the study was to test the psychometric equivalence and validate the Greek translation of the long version of the Copenhagen Psychosocial Questionnaire (COPSOQ III-GR) in a sample of Greek employees (*n* > 2000). The study sample comprised 2189 participants who completed all the survey questions [45]. For the development and conduct of the COPSOQ III-GR study, the authors received approval from the International COPSOQ Network.

### 2.3. The Greek Version of COPSOQ III

The overall design and content of the new Greek version (III) of the questionnaire was based on a scientific collaboration outcome between the Hellenic COPSOQ Research Team (University of Piraeus, Greece) and the Steering Committee of the International COPSOQ-Research Network (since 2017, October). The overall development process of the Greek COPSOQ ΙΙΙ was carried out in three phases. In the first phase, the Greek version III instrument was constructed (based on the German COPSOQ-III scales and including a few add-on scales from the COPSOQ-II scales). In the second phase, forward–backwards translation and cultural adaptation of the COPSOQ-III (GR) questionnaire were performed. In the third phase, the psychometric properties of the new COPSOQ-III (GR) version were evaluated in several cross-sectoral and cross-occupational samples of Greek employees (*n* = 2189). The measurement qualities of the Greek COPSOQ III have been explored in accordance with the usual requirements of a validation study, as defined by DIN EN ISO 10075-3 [46]. Permission to design and validate the Greek III version of the diagnostic tool was obtained by the Steering Committee of the International COPSOQ network in the context of the academic research activities of the School of Economics, Business and International Studies at the University of Piraeus, Greece.

The core version of the Greek COPSOQ-III (core) included a total of 108 items and 21 core scales. Answers to all the questions were given through Likert-type responses. It has been designed under a job demands–control and domain-centric approach and is based on the COPSOQ German-III version [39]. The first pilot study (*n* = 426) used the first core scales (21 scales). The findings of the pilot study were presented in the Employability for the 21st Century International Conference Proceedings in Belgium [43]. Within 2019, the Greek version was re-engineered and finalized to support international comparisons and benchmarking. Today, it is available in two official releases for Greece and Cyprus (Language: GR), the COPSOQ III-GR Long Version (108 items, 40 scales) and the COPSOQ-III-GR Middle Version (74 items, 24 scales) (see Domains and Items per scale in Table S1 in the Supplementary Materials). We have used the COPSOQ III-GR Long Version in this study to obtain a validation reference for all items included in the Greek language questionnaire.

The new “long” version of the COPSOQ III (GR) questionnaire was shared in two different ways (internet-cloud and in writing). With respect to the companion letter–paper documentation for the COPSOQ-III (GR) questionnaire, the participant-consent form, the relevant cover letter explaining the purpose of the research study, and the researchers’ affiliation were enclosed in the same envelope. The full paper set was handed over to employees who belonged to many different occupational sectors. In the case of a company or an organizational entity having concerns about the participation of their employees in the study, written approval from the scientific committee or the HR department of the company was an additional prerequisite of our study. Regarding the “internet-cloud version” of the questionnaire, two types of internet-cloud-based applications were selected during the overall survey-feedback collection process. The first application was a secure cloud-interface application based on Google Forms. The second application was a Microsoft Azure application, named Academic Research Radar (ARR), which is a cloud platform that supports specific parameterizing, building, distributing, and administering online surveys under top-security and confidentiality specifications (powered MS-Azure-specific services and overall data management). As an original Azure app, the ARR cloud platform took full advantage of the “Microsoft Azure Compliance Manager” solution. The Azure Compliance Manager is a free Microsoft cloud services solution designed to help organizations meet complex compliance obligations, including the GDPR, ISO 27001 [47], ISO 27018 [48], and NIST 800-53 [49], with the aim of helping the academic research community assess, implement, and manage Azure security policies and GDPR compliance-related policies from within Microsoft Azure Cloud applications. The ARR platform is used by academic staff and researchers to collect any type of “voice of the employee” feedback via a secure online cloud interface powered by Azure GDPR compliance features. In our study, the ARR Azure application was parameterized to support COPSOQ-III (GR) academic surveys in terms of confidentiality, security, participants’ anonymity, organizational-entity anonymity, and GDPR compliance at the national level. Thus, both types of electronic participation—Google forms and the Microsoft Azure App—were two fully anonymous and confidential user interfaces (for the participants) in terms of electronic form fill-in, data storage, confidentiality, compliance, and overall survey data processing.

### 2.4. Statistical Analyses

Statistical analyses were performed via Statistical Software R (version 4.2.0, Vienna, Austria). Cronbach’s α and the intraclass correlation coefficient (ICC) were computed to assess the reliability and homogeneity of the scales in the sample. We used the Cronbach-Alpha function from the ltm package (version 1.2, Rotterdam, The Netherlands) [50] to calculate the Cronbach’s α and the ICC function from the irr package (version 0.84.1, Münster, Germany) [51]. To explore the characteristics of each scale’s distribution, we calculated the mean, standard deviation, and floor and ceiling effects via base R functions (e.g., mean, sd). The floor effect is defined as the percentage of answers coded zero, whereas the ceiling effect is defined as the percentage of answers coded 100. We also assessed the internal validity and distinctiveness of the scales via Pearson’s correlations and multivariable relationships via explorative factor analysis (EFA) and generalized linear regression models. For Pearson correlations and generalized linear regression, we used the cor and glm functions, respectively, from the basic R stats package. For the EFA, we used the EFA function from the EFAtools package (version 0.4.4, Austin, USA) [52] via the rotation method (varimax with Kaiser normalization; eigenvalue of at least 1 as the criterion). Statistical significance was considered at the level of <0.05. Bonferroni corrections were applied in multivariable regression models to take account of multiple testing (0.05/34 psychosocial work factors ≈ 0.0015).

## 3. Results

The sociodemographic and occupational characteristics of the sample are presented in Table 1. From a sociodemographic perspective, 49.5% of the participants in the sample were female, and 50.5% were male. In terms of age, 31.7% of the participants were up to 24 years of age, 17.2% were between the ages of 25 and 34 years, 30.7%, 17.2%, and 3.2% were between the ages of 35 and 44 years, 45 to 54 years, and 55 years or older, respectively. A total of 43.6% of the participants were working in the public sector, whereas 56.4% were working in the private sector. The largest percentage of employees (25.9%) were working in “admin, not leading” occupations, and the smallest percentage (2.6%) were working in “tech: engineers” occupations (see Table S2 in the Supplementary Materials for the mappings of the occupational categories and the ISCO-08 codes). In terms of interaction with external clients, 42.2% of the participants stated that they did not have any interaction, 4.7% had, on average, less than one interaction per day, 6.9% had one to five interactions per day, 10.6% had six to 14 interactions per day, and 35.6% had 15 or more interactions per day. Finally, 21.5% of the participants were supervisors, whereas 61% were not supervisors in jobs with supervisors, and 17.5% were not supervisors in jobs with no supervisors (e.g., freelancers).

The 40 scales of the Greek COPSOQ III questionnaire are presented in Table 2. The means, standard deviations, and fractions with ceiling and floor effects were calculated for each scale to assess sensitivity and variation. The mean values of the scales varied from 0.3 for “Physical Violence” to 71.55 for “Work Pace”. The standard deviations of all scales ranged from a minimum of 3.1 points to a maximum of 38.96 points. Floor effects ranged between 48% and 99.18%. There were four scales with scores of 90% or more in this category (“Cyber Bullying”, “Physical Violence”, “Sexual Harassment”, “Threats of Violence”), whereas three scales had scores of less than 10% (“Influence”, “Variation of Work”, “Work Pace”). The ceiling effects ranged between 0% and 55.29%. In addition, there were four scales exceeding 20% (“Bullying from Customers (External)”, “Demands for Hiding Emotions”, “Influence”, “Mobbing”), whereas 22 scales provided fewer than 5% answers in this extreme category. Table 2 also shows the Cronbach’s alpha and the intraclass correlation (ICC) for each scale. There is a broad consensus that a value of α ≥ 0.7 is an indicator of acceptable reliability, and a value of ICC ≥ 0.5 is an indicator of an acceptable degree of congruence. In total, 22 scales showed good reliability in relation to Cronbach’s alpha, and 16 scales showed good homogeneity in terms of the ICC.

**Figure 1:**
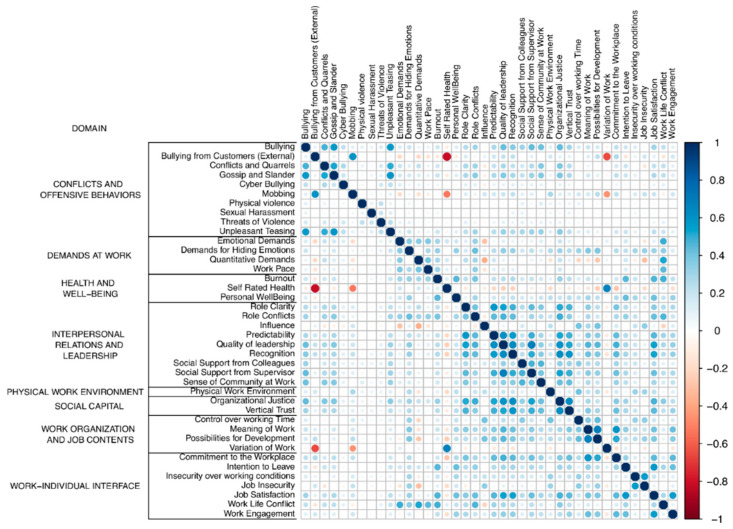
Pairwise Pearson correlations. The 40 scales are classified into eight domains.

In Figure 1, we present the pairwise correlation coefficients (Pearson’s r) as indicators of the internal validity and distinctiveness of the scales. Usually, if “r” is lower than |0.1|, the correlation is said to be negligible. Values between |0.1| and |0.3| are considered weak correlations, values between |0.3| and |0.5| are considered moderate correlations, and values greater than |0.5| are interpreted as strong correlations. In this sense, out of a total of 780 pairwise correlations, “r” was weak in 613 cases (78.6%), moderate in 133 cases (17.1%), and strong in 34 cases (4.4%), with −0.81 being the strongest correlation (between “Self-Rated Health” and “Bullying from Customers (External)”).

Exploratory factor analysis was used to assess the statistical relationships for a multitude of scales. In Table 3 and Table 4, we delineate the results of the EFA performed by treating workplace factors and effects separately in accordance with the generalized model of cause and effect. In both tables, all factor loadings lower than |0.4| are hidden for better readability. In Table 3, components were extracted from the 34 psychosocial work factors, with the sum of the squared loadings explaining 44.8% of the total variance. Table 4 shows that out of the six scales of effects, two components were extracted, covering 48.0% of the total variance.

Finally, we applied six multiple linear regression models. The satisfaction and health scales are each defined as outcome (dependent) variables to be predicted by the 34 work factors. All regression estimates and standard errors are presented in Table 5, with the star symbols indicating the statistical significance of each estimate and the rhombus symbols representing the statistical significance after Bonferroni corrections. Table 6 shows the variance explained (determination coefficient R^2^) by a model including all 34 workplace factors as independent variables (i.e., full model) and by a model including only the top five factors selected as those having the lowest *p* values (i.e., most statistically significant variables) in the full model. Table 6 also shows the Akaike Information Criterion (AIC) and the degrees of freedom (df) for each model. Multiple linear regression models applied also in subgroup analyses separated by public and private sectors (see Figure S1A,B in the Supplementary Materials) and by eight categories of occupations (see Figure S2A–H in the Supplementary Materials).

Burnout was much better predicted than all other dependent variables (R^2^ = 0.905), which means that 90.5% of its variance was explained by the model. The top five predictors for “Burnout” were “Work Life Conflict”, “Work Pace”, “Job Insecurity”, “Quantitative Demands” and “Role Conflicts”. Consequently, “Job Satisfaction” (R^2^ = 0.876), was followed by “Work Engagement” (R^2^ = 0.806), “Self-Rated Health” (R^2^ = 0.795), “Personal Well-being” (R^2^ = 0.718), and “Intention to leave” (R^2^ = 0.564). The top five predictors for “Job Satisfaction” were “Recognition”, “Quality of Leadership”, “Commitment to the Workplace”, “Role Conflicts” and “Physical Work Environment”. The top five predictors for “Work Engagement” were “Commitment to the Workplace”, “Meaning of Work”, “Influence”, “Bullying”, and “Vertical Trust”. The top five predictors for “Self-Rated Health” were “Variation of Work”, “Bullying from Customers (External)”, “Work Pace”, “Influence” and “Social Support from Colleagues”. The top five predictors for “Personal Well-being” were “Bullying from Customers (External)”, “Insecurity over Working Conditions”, “Commitment to the Workplace”, “Role Conflicts”, and “Role Clarity”. Finally, the top five predictors for “Intention to Leave” were “Bullying from Customers (External)”, “Commitment to the Workplace”, “Meaning of Work”, “Role Conflicts” and “Job Insecurity”.

## 4. Discussion

In summary, our validation study of the Greek COPSOQ III version showed adequate reliability and validity, which is in line with the validation of the COPSOQ III questionnaire from other European countries. For example, our findings are compatible with the validation of the German COPSOQ III, which represented the original source of the corresponding Greek questionnaire [39], finding either good or very good reliability and validity for most of their 84 items and 31 scales. On the basis of the statistical analyses and findings of the current study, there is substantial evidence that the top work-environment predictor for burnout syndrome-related risk in Greece is an imbalance between work-life and family-life. The top predictor for “Self-rated Health”, “Personal Wellbeing” and “Intention to Leave” related risk is “External Bullying” (Bullying from Customers). Thus, “Bullying from Customers” is a common predictor for all three of the above psychosocial effects on Greek employees. In addition, the top predictor for “Work-Engagement” (lack of work-engagement) is the work-environment factor “Commitment to the Workplace” (lack of commitment to the workplace). Finally, the top predictor for the lack of “Job Satisfaction” risk is a lack of “Recognition”. In more detail, our regression analyses also revealed that 34 psychosocial workplace factors can predict, with good accuracy, the scores of job satisfaction and health scales. First, “Burnout” was predicted much better than all other effects; next was “Job Satisfaction”, followed by “Work Engagement”, “Self-Rated Health”, “Personal Wellbeing”, and “Intention to Leave”. The analysis also revealed the top five predictors for each outcome variable. The top five predictors for “Self-Rated Health” are “Variation of Work”, “Bullying from Customers (External)”, “Work Pace”, “Influence” and “Social Support from Colleagues”.

Predictors for “Intention to Leave” included “Bullying from Customers (External)”, “Commitment to the Workplace”, “Meaning of Work”, “Role Conflicts” and “Job Insecurity”. As nurses account for the largest number of workers in most healthcare systems, these factors play an important role [53]. Different studies agree with our results that occupational factors such as poor managerial support, lack of meaning of work, role conflicts, lack of opportunities for job promotion, job stress, and work–reward imbalance can be associated with employees’ intention to leave [54,55]. Another study has shown that a supportive work climate is a predictor for retaining in the nursing profession [56]. This is in line with our study in which we had participants from different categories of health professionals including nurses (included in the category of other professions).

The top five predictors for “Job Satisfaction” were “Recognition”, “Quality of Leadership”, “Commitment to the Workplace”, “Role Conflicts”, and “Physical Work Environment”. The literature review has shown that nurses’ job satisfaction predictors include working conditions, relationships with coworkers and leaders, pay, promotion, security of employment, responsibility, and working hours [57,58]. The top five predictors for “Personal Well-being” were “Bullying from Customers (External)”, “Insecurity over Working Conditions”, “Commitment to the Workplace”, “Role Conflicts”, and “Role Clarity”.

The most important predictors for “Burnout” were “Work Life Conflict”, “Work Pace”, “Job Insecurity”, “Quantitative Demands” and “Role Conflicts”. For example, research consistently shows that adverse job characteristics such as high workload, low staffing levels, long shifts, low control, low schedule flexibility, time pressure, high job and psychological demands, low task variety, role conflict, low autonomy, negative nurse—physician relationships, poor supervisor/leader support, poor leadership, negative team relationships, and job insecurity are associated with burnout in nursing [59].

The top five predictors for “Work Engagement” were “Commitment to the Workplace”, “Meaning of Work”, “Influence”, “Bullying”, and “Vertical Trust”. Work engagement is a positive, fulfilling state of mind about work that is characterized by vigour, dedication, and absorption. Trust (organizational, managerial, and collegiality) and autonomy are the antecedents of work engagement [60]. In nursing, work engagement is a dedicated, absorbing, vigorous nursing practice that emerges from settings of autonomy and trust and results in safer, cost-effective patient outcomes. From this definition, work engagement can be developed as an explanatory middle range theory that conceptually captures the concerns that health professionals have about their work environment. The assumptions that underlie work engagement, the linkages between the antecedents of autonomy and trust, and the relationships between the antecedents of trust and autonomy and the closely related concepts of transformational and authentic leadership styles are some of the remaining areas to be developed in the middle range theory.

Our study is the first to be conducted in Greece to validate the COPSOQ III Greek version of this internationally applied tool; nevertheless, several limitations of our study should be acknowledged. We believe that the study sample (*n* = 2189) was adequate, and that the internal consistency of the subscales was satisfactory. Although our study included employees from a variety of occupational sectors and different types of workplaces in Greece, we were not able to perform sector analyses to assess possible differences in the performance of the tool. Nevertheless, we are presenting Forest Plots in the Supplementary Materials with estimated coefficients and 95% confidence intervals for the psychosocial work factors regressed with self-rated health, intention to leave, job satisfaction, personal well-being, burnout and work engagement in subgroup analyses for the eight job categories included in Table 1. At the same time, we acknowledge that we were not able to analyze specific occupational sectors, limiting the interpretation of our findings. We recognize our conveniently chosen sample, which precludes the generalization of our findings. In addition, during the study, we did not perform “test-retest” reliability because we distributed the GR-Long version of the COPSOQ-III questionnaire (108 items, 40 scales); however, future research is planned to address this psychometric property. While the overall reliability and statistical robustness of many of the scales is acceptable, certain scales exhibit low internal consistency (e.g., control over working time, emotional demands) or are limited by single-item construction, making reliability assessment impossible. In addition, some scales (e.g., cyberbullying, physical violence) show extreme floor effects, which may reduce their utility in general population settings.

In summary, the COPSOQ III offers a comprehensive framework for assessing psychosocial factors in the workplace, making it a valuable tool for developing effective public health strategies. It encompasses a wide range of theoretical approaches and delivers in-depth information on psychosocial working conditions, supporting evidence-based organizational research and diagnosis. Moreover, it facilitates advanced psychosocial risk management during organizational change, benefiting both public and private sector organizations. The Greek validation study of the COPSOQ III highlights its utility in identifying key psychosocial stressors, such as work—life conflict, bullying, job insecurity, and role conflicts, which are significant predictors of mental health outcomes such as burnout and job satisfaction. By incorporating the COPSOQ III into public health strategies, policymakers and health professionals can better understand and address the psychosocial dimensions of occupational health. For example, the results from the COPSOQ III study can inform targeted interventions aimed at reducing work-related stress and enhancing employee well-being. Public health initiatives can leverage these data to implement supportive policies, such as flexible work arrangements, antibullying programs, and leadership training, which are critical in mitigating the adverse effects of psychosocial stressors [61]. Additionally, the COPSOQ III can serve as a tool for ongoing monitoring and evaluation, ensuring that interventions remain effective and responsive to emerging psychosocial risks. In the context of broader public health challenges, the COPSOQ III Greek validation study aligns with findings from other research on mental health and workplace adaptations post-pandemic. Additionally, researchers [2,62] emphasize the importance of addressing mental health comprehensively. These studies highlight the need for robust psychosocial assessment tools such as the COPSOQ III to support mental health initiatives and policy interventions. Furthermore, integrating COPSOQ III into public health strategies can increase the effectiveness of clinical interventions for mental health disorders in the context of psychotic spectrum disorders and bipolar disorder [63]. By identifying workplace-related psychosocial risks, public health strategies can be tailored to address specific stressors that exacerbate mental health issues, thus improving overall treatment outcomes [64,65,66].

Future research could investigate cognitive-based interventions aimed at enhancing workplace cognitive functioning and alleviating stress. A deeper understanding of the neuropsychological mechanisms through which workplace stress impairs cognitive performance and mental health would offer valuable insights. Additionally, exploring the protective role of family support in buffering the effects of high job demands on employee well-being could further enrich this area of study. Comprehensive intervention strategies targeting workplace bullying and harassment should also be developed and evaluated, incorporating the psychosocial dimensions identified by the COPSOQ III questionnaire. Finally, examining the impact of recent legislative reforms on the prevalence and management of workplace bullying and harassment in Greece could yield practical, policy-relevant findings.

## 5. Conclusions

In conclusion, the Greek adaptation of the COPSOQ III (GR) demonstrated satisfactory psychometric robustness across the majority of its scales, aligning with internationally recognized standards for instrument validation. These findings support its applicability as a reliable and valid tool for both empirical research and the systematic assessment of psychosocial risks across diverse organizational settings in the public and private sectors.

## Figures and Tables

**Table 1:** Study sample: sociodemographic and occupational characteristics.

Feature	Sample of COPSOQ-Database (n=2189)		
	Category	Frequency	Percentage
Gender	Male	1105	50.5
	Female	1084	49.5
Age groups	up to 24	693	31.7
	25–34	377	17.2
	35–44	673	30.7
	45–54	376	17.2
	55 and more	70	3.2
Supervisor	No (jobs with no supervisors)	384	17.5
	Yes	470	21.5
	No (jobs with supervisors)	1335	61.0
Interface with external clients	No, never	923	42.2
	Yes, less than 1 time per day (on average)	103	4.7
	Yes, 1 to 5 times per day (on average)	151	6.9
	Yes, 6 to 14 times per day (on average)	233	10.6
	Yes, 15 or more times per day (average)	779	35.6
Sector	Public	954	43.6
	Private	1235	56.4
Profession ^a^	Admin, leading	336	15.3
	Tech, leading	190	8.7
	Workers	202	9.2
	Tech: engineers	56	2.6
	Tech: technicians	274	12.5
	Admin, not leading	567	25.9
	Tech, not leading	97	4.4
	Other professions.	467	21.3

^a^ See Table S2 in the Supplementary Materials for the mappings of the occupational categories and the ISCO-08 codes.

**Table 2:** Descriptive statistics and reliability of the scales.

Scale	*n*	*a*	ICC	M	SD	Floor Effect (%)	Ceiling Effect (%)
Burnout	5	0.73	0.32	43.64	26.71	12.41	5.24
Bullying	2	0.94	0.87	13.47	20.52	61.06	1.1
Bullying from Customers (External)	9	0.96	0.68	64.87	38.96	16.33	46.34
Role Clarity	3	0.81	0.53	20.1	21.94	43	1.48
Role Conflicts	3	0.82	0.6	49.38	29.02	13.11	10.43
Conflicts and Quarrels	1	-	-	10.27	17.23	67.98	0.23
Control over Working Time	2	0.49	0.3	38.01	29.55	18.82	10
Commitment to the Workplace	2	0.68	0.3	33.22	30.49	32.09	7.51
Emotional Demands	2	0.57	0.09	36.45	33.22	35.38	7.72
Self-Rated Health	1	-	-	29.56	31.75	23.98	4.93
Gossip and Slander	1	-	-	18.73	24.18	50.53	2.1
Demands for Hiding Emotions	2	0.73	0.5	57.47	32.54	12.79	22.09
Cyber Bullying	1	-	-	0.9	6.1	97.26	0.05
Influence	3	0.8	0.55	63.32	28.14	3.33	25.17
Intention to Leave	2	0.81	0.68	16.21	22.79	55	2.49
Insecurity over Working Conditions	3	0.65	0.28	32.22	32.45	37.43	8.82
Job Insecurity	3	0.8	0.56	47.99	34.38	21.08	17.36
Job Satisfaction	7	0.88	0.47	33.35	24.18	18.67	3
Organizational Justice	2	0.72	0.56	37.6	25.29	15.19	4.29
Mobbing	5	0.99	0.96	58.38	46.47	35.79	55.29
Meaning of Work	2	0.89	0.78	25.23	26.31	38.97	3.63
Possibilities for Development	2	0.84	0.72	41.83	31.56	20.05	12.43
Predictability	2	0.68	0.34	42.1	29.49	16.49	9.48
Physical Violence	1	-	-	0.3	3.53	99.18	0
Personal Well-being	6	0.87	0.47	24.96	26.89	43.17	2.22
Physical Work Environment	6	0.83	0.4	36.62	35.85	37.87	11.69
Quantitative Demands	3	0.79	0.52	34	27.19	26.13	2.68
Quality of Leadership	4	0.87	0.56	43.18	29.97	15.63	11.58
Recognition	1	-	-	40.81	28.81	16.26	8.59
Social Support from Colleagues	3	0.41	0.15	31.37	26.76	26.33	4.84
Sexual Harassment	1	-	-	0.32	3.1	98.81	0
Social Support from Supervisor	4	0.76	0.37	30.38	28.25	31.43	5.11
Sense of Community at Work	2	0.7	0.54	13.62	17.07	54.59	0.34
Vertical Trust	2	0.64	0.47	27.07	22.22	26.79	1.69
Threats of Violence	1	-	-	0.7	4.7	97.58	0
Unpleasant Teasing	1	-	-	9.86	18.58	71.63	0.73
Variation of Work	1	-	-	10.31	18.25	4.39	0.59
Work Life Conflict	7	0.84	0.34	34.65	33.35	37.34	8.61
Work Engagement	3	0.69	0.36	27.25	22.43	26.62	1.37
Work Pace	2	0.82	0.68	71.55	18.65	0.48	17.95

**Table 3:** EFA of psychosocial work factors: rotated factor matrix.

Psychosocial Work Factors ^a^	Factor Loading ^b^				
	1	2	3	4	5
Bullying	0.32		0.68		
Bullying from Customers (External)					0.85
Role Clarity	0.67				
Role Conflicts	0.41			0.53	
Conflicts and Quarrels			0.60		
Control over Working Time		0.63			
Commitment to the Workplace	0.62				
Emotional Demands				0.60	
Gossip and Slander			0.64		
Demands for Hiding Emotions		0.51		0.40	
Cyber Bullying			0.31		
Influence		0.52		0.36	
Insecurity over Working Conditions		0.53			
Job Insecurity		0.67			
Organizational Justice	0.67				
Mobbing				0.61	
Meaning of Work	0.58	0.45			
Possibilities for Development	0.51	0.56			
Predictability	0.68				
Physical Violence					
Physical Work Environment		0.50			
Quantitative Demands		−0.39		0.58	
Quality of Leadership	0.75				
Recognition	0.69				
Social Support from Colleagues	0.39		0.31		
Sexual Harassment					
Social Support from Supervisor	0.67				
Sense of Community at Work	0.48		0.39		
Vertical Trust	0.67				
Threats of Violence					
Unpleasant Teasing			0.74		
Variation of Work					−0.71
WorkLife Conflict				0.71	
Work Pace				0.59	

^a^ Eigenvalue ≥ 1, total variance explained 44.8%. ^b^ Only loadings ≥ |0.30| are shown.

**Table 4:** EFA on effects: rotated factor matrix.

Effects ^a^	Factor Loading ^b^	
	1	2
Self-Rated Health		−0.55
Intention to Leave	0.70	
Job Satisfaction	0.73	0.47
Personal Well-being	0.62	
Burnout	0.69	
Work Engagement	0.51	0.44

^a^ Eigenvalue ≥ 1, total variance explained 48.0%. ^b^ Only loadings ≥ |0.40| are shown.

**Table 5:** Estimates and standard errors of the models where each outcome variable is regressed on all 34 psychosocial workplace factors.

	Outcome	Self-Rated Health	Intention to Leave	Job Satisfaction	Personal Well-Being	Burnout	Work Engagement
Exposure							
Bullying		−0.022 (0.031)	−0.028 (0.027)	0.012 (0.021)	−0.023 (0.027)	−0.034 (0.023)	−0.086 (0.023) *♦
Bullying from Customers (External)		−0.341 (0.018) *♦	−0.120 (0.016) *♦	−0.028 (0.012) *	−0.116 (0.016) *♦	−0.039 (0.014) *	0.048 (0.013) *♦
Role Clarity		0.068 (0.033) *	−0.044 (0.030)	−0.060 (0.023) *	−0.143 (0.029) *♦	0.025 (0.025)	0.001 (0.025)
Role Conflicts		0.019 (0.024)	0.117 (0.021) *♦	0.079 (0.016) *♦	0.109 (0.021) *♦	0.082 (0.018) *♦	0.039 (0.018) *
Conflicts and Quarrels		0.021 (0.033)	0.052 (0.029)	0.025 (0.022)	0.025 (0.029)	0.039 (0.025)	0.039 (0.024)
Control over Working Time		−0.032 (0.024)	−0.018 (0.021)	−0.047 (0.016) *	0.011 (0.021)	0.012 (0.018)	−0.013 (0.018)
Commitment to the Workplace		−0.067 (0.024) *	0.141 (0.022) *♦	0.086 (0.016) *♦	0.114 (0.021) *♦	0.031 (0.018)	0.135 (0.018) *♦
Emotional Demands		0.072 (0.022) *♦	0.017 (0.020)	−0.005 (0.015)	0.086 (0.020) *♦	0.039 (0.017) *	−0.004 (0.017)
Gossip and Slander		−0.059 (0.025) *	−0.010 (0.022)	0.011 (0.017)	0.001 (0.022)	0.021 (0.019)	−0.017 (0.018)
Demands for Hiding Emotions		0.041 (0.021)	0.005 (0.019)	0.007 (0.014)	0.063 (0.018) *♦	0.027 (0.016)	−0.020 (0.015)
Cyber Bullying		−0.078 (0.075)	−0.126 (0.067)	0.048 (0.051)	0.027 (0.066)	−0.030 (0.057)	0.001 (0.055)
Influence		0.191 (0.021) *♦	0.021 (0.019)	0.041 (0.014) *	0.033 (0.018)	0.059 (0.016) *♦	0.073 (0.016) *♦
Insecurity over Working Conditions		−0.038 (0.024)	0.077 (0.021) *♦	0.015 (0.016)	0.125 (0.021) *♦	0.029 (0.018)	−0.024 (0.018)
Job Insecurity		0.040 (0.021)	−0.070 (0.018) *♦	−0.009 (0.014)	0.018 (0.018)	0.082 (0.016) *♦	0.038 (0.015) *
Organizational Justice		0.057 (0.031)	0.006 (0.028)	0.057 (0.021) *	−0.001 (0.027)	0.056 (0.023) *	−0.011 (0.023)
Mobbing		−0.021 (0.012)	0.026 (0.011) *	0.037 (0.008) *♦	0.026 (0.011) *	0.019 (0.009) *	0.018 (0.009) *
Meaning of Work		−0.002 (0.027)	0.148 (0.024) *♦	0.028 (0.018)	0.082 (0.023) *♦	0.023 (0.020)	0.121 (0.020) *♦
Possibilities for Development		−0.093 (0.023) *♦	0.020 (0.021)	0.046 (0.016) *	−0.017 (0.020)	−0.012 (0.018)	0.028 (0.017)
Predictability		0.000 (0.026)	−0.021 (0.023)	0.042 (0.018) *	0.038 (0.023)	−0.033 (0.020)	0.009 (0.019)
Physical violence		−0.168 (0.126)	−0.304 (0.113) *	0.008 (0.086)	−0.040 (0.111)	0.042 (0.095)	0.116 (0.094)
Physical Work Environment		0.006 (0.020)	−0.026 (0.018)	0.065 (0.014) *♦	0.077 (0.018) *♦	0.007 (0.015)	0.015 (0.015)
Quantitative Demands		0.069 (0.027) *	0.081 (0.024) *♦	0.019 (0.018)	−0.025 (0.023)	0.095 (0.020) *♦	0.053 (0.020) *
Quality of Leadership		−0.115 (0.031) *♦	−0.023 (0.027)	0.115 (0.021) *♦	−0.030 (0.027)	−0.036 (0.023)	0.014 (0.023)
Recognition		−0.038 (0.023)	0.064 (0.020) *	0.095 (0.016) *♦	0.013 (0.020)	0.032 (0.017)	0.029 (0.017)
Social Support from Colleagues		0.162 (0.032) *♦	0.016 (0.028)	0.031 (0.021)	0.026 (0.028)	0.067 (0.024) *	−0.051 (0.023) *
Sexual Harassment		−0.074 (0.143)	−0.087 (0.128)	−0.027 (0.097)	−0.013 (0.125)	0.197 (0.108)	−0.141 (0.106)
Social Support from Supervisor		0.056 (0.032)	0.043 (0.029)	0.004 (0.022)	−0.063 (0.028) *	0.004 (0.024)	0.041 (0.024)
Sense of Community at Work		−0.054 (0.037)	0.013 (0.033)	0.028 (0.025)	0.119 (0.033) *♦	−0.003 (0.028)	0.098 (0.028) *♦
Vertical Trust		0.050 (0.032)	0.030 (0.029)	0.054 (0.022) *	0.105 (0.028) *♦	0.033 (0.024)	0.087 (0.024) *♦
Threats of Violence		−0.059 (0.099)	0.063 (0.089)	0.018 (0.068)	−0.121 (0.087)	−0.084 (0.075)	−0.127 (0.074)
Unpleasant Teasing		−0.019 (0.032)	0.081 (0.029) *	−0.035 (0.022)	−0.019 (0.028)	0.002 (0.025)	0.025 (0.024)
Variation of Work		0.706 (0.030) *♦	0.056 (0.026) *	0.037 (0.020)	−0.045 (0.026)	0.026 (0.022)	0.020 (0.022)
WorkLife Conflict		0.018 (0.028)	0.063 (0.025) *	0.048 (0.019) *	0.031 (0.024)	0.207 (0.021) *♦	0.015 (0.021)
Work Pace		0.376 (0.025) *♦	−0.019 (0.023)	0.035 (0.017) *	0.034 (0.022)	0.171 (0.019) *♦	0.012 (0.019)
R^2^		0.795	0.558	0.874	0.711	0.901	0.794
R^2^ adjusted		0.791	0.551	0.872	0.706	0.899	0.790

* denotes a *p*-value < 0.05; **♦** denotes a *p* value < 0.05/34 ≈ 0.0015.

**Table 6:** Model fit parameters and the top five predictors for each dependent variable.

Dependent Scale	Total Model Fit		Model Fit with Top five ^a^ Predictors		Top Five ^a^ Predictors	Estimated Coefficient (Std Error) ^b^
	R^2^	AIC (df)	R^2^	AIC (df)		
Self-Rated Health	0.795	13,107.96 (34)	0.777	13,229.95 (5)	Variation of Work Bullying from Customers (External) Work Pace Influence Social Support from Colleagues	0.744 (0.030) −0.385 (0.015) 0.487 (0.017) 0.120 (0.018) 0.089 (0.025)
Intention to Leave	0.564	12,599.47 (40)	0.524	12,723.22 (5)	Bullying from Customers (External) Commitment to the Workplace Meaning of Work Role Conflicts Job Insecurity	−0.106 (0.011) 0.195 (0.021) 0.157 (0.020) 0.256 (0.014) −0.016 (0.013)
Job Satisfaction	0.876	11,411.47 (40)	0.861	11,598.03 (5)	Recognition Quality of Leadership Commitment to the Workplace Role Conflicts Physical Work Environment	0.140 (0.014) 0.200 (0.017) 0.150 (0.014) 0.177 (0.013) 0.109 (0.012)
Personal Well-being	0.718	12,491.16 (40)	0.673	12,745.61 (5)	Bullying from Customers (External) Insecurity over Working Conditions Commitment to the Workplace Role Conflicts Role Clarity	−0.046 (0.010) 0.249 (0.017) 0.226 (0.018) 0.270 (0.015) −0.112 (0.025)
Burnout	0.905	11,798.87 (40)	0.892	12,013.86 (5)	Work Life Conflict Work Pace Job Insecurity Quantitative Demands Role Conflicts	0.244 (0.020) 0.255 (0.015) 0.130 (0.011) 0.074 (0.019) 0.153 (0.015)
Work Engagement	0.806	11,681.94 (40)	0.774	11,943.91 (5)	Commitment to the Workplace Meaning of Work Influence Bullying Vertical Trust	0.213 (0.017) 0.116 (0.017) 0.165 (0.009) −0.005 (0.017) 0.206 (0.019)

^a^ Top predictors are the first five workplace factors with the lowest *p* values in the full model. ^b^ Model with the top five predictors.

## Data Availability

The original data presented in the study are openly available in Zenodo Repository at https://zenodo.org/records/16575662 (accessed on 10 July 2025).

## Supplementary Materials

The following supporting information can be downloaded at: https://www.mdpi.com/article/10.3390/healthcare13161980/s1, Table S1: Domains & Items per scale in long and middle versions of Greek COPSOQ-III questionnaire; Table S2: Occupation Categories and their ISCO-08 mappings; Figure S1A: Estimated coefficients and 95% confidence intervals for the psychosocial work factors regressed with self-rated health, intention to leave, job satisfaction, personal wellbeing, burnout and work engagement in a subgroup analysis for the Public sector; Figure S1B: Estimated coefficients and 95% confidence intervals for the psychosocial work factors regressed with self-rated health, intention to leave, job satisfaction, personal wellbeing, burnout and work engagement in a subgroup analysis for the Private sector; Figure S2A: Estimated coefficients and 95% confidence intervals for the psychosocial work factors regressed with self-rated health, intention to leave, job satisfaction, personal wellbeing, burnout and work engagement in a subgroup analysis for Admin, leading; Figure S2B: Estimated coefficients and 95% confidence intervals for the psychosocial work factors regressed with self-rated health, intention to leave, job satisfaction, personal wellbeing, burnout and work engagement in a subgroup analysis for Tech, leading; Figure S2C: Estimated coefficients and 95% confidence intervals for the psychosocial work factors regressed with self-rated health, intention to leave, job satisfaction, personal wellbeing, burnout and work engagement in a subgroup analysis for Workers; Figure S2D: Estimated coefficients and 95% confidence intervals for the psychosocial work factors regressed with self-rated health, intention to leave, job satisfaction, personal wellbeing, burnout and work engagement in a subgroup analysis for Tech: engineers; Figure S2E: Estimated coefficients and 95% confidence intervals for the psychosocial work factors regressed with self-rated health, intention to leave, job satisfaction, personal wellbeing, burnout and work engagement in a subgroup analysis for Tech: technicians; Figure S2F: Estimated coefficients and 95% confidence intervals for the psychosocial work factors regressed with self-rated health, intention to leave, job satisfaction, personal wellbeing, burnout and work engagement in a subgroup analysis for Admin, not leading; Figure S2G: Estimated coefficients and 95% confidence intervals for the psychosocial work factors regressed with self-rated health, intention to leave, job satisfaction, personal wellbeing, burnout and work engagement in a subgroup analysis for Tech, not leading; Figure S2H: Estimated coefficients and 95% confidence intervals for the psychosocial work factors regressed with self-rated health, intention to leave, job satisfaction, personal wellbeing, burnout and work engagement in a subgroup analysis for Other professionals.
