# Supplementary material for: A Validation Study of the COPSOQ III Greek Questionnaire for Assessing Psychosocial Factors in the Workplace
Source: Healthcare (Basel). 2025 Aug 12;13(16):1980. doi: 10.3390/healthcare13161980 (PMC12385713; doi:10.3390/healthcare13161980)
Supplement: Supplementary file 1 [file healthcare-13-01980-s001.zip › healthcare-3744069-supplementary.pdf]

Table S1: Domains & Items per scale in long and middle versions of Greek COPSOQ-III questionnaire

GR-DOMAIN	GR-SCALE	Number of Questions (GR-LONG)	SCALE	Number of Questions (GR-MIDDLE)
CONFLICTS AND OFFENSIVE BEHAVIORS	Bullying	2	BU	2
CONFLICTS AND OFFENSIVE BEHAVIORS	Bullying from Customers (External)	8	BUEXT	8
CONFLICTS AND OFFENSIVE BEHAVIORS	Conflicts and Quarrels	1	CQ	_
CONFLICTS AND OFFENSIVE BEHAVIORS	Cyber Bullying	1	HSM	_
CONFLICTS AND OFFENSIVE BEHAVIORS	Gossip and Slander	1	GS	_
CONFLICTS AND OFFENSIVE BEHAVIORS	Sexual Harassment	1	SH	1
HEALTH AND WELL-BEING	Burnout	5	BO	5
HEALTH AND WELL-BEING	Personal WellBeing	5	PWB	5
HEALTH AND WELL-BEING	Self Rated Health	1	GH	1
WORK-INDIVIDUAL INTERFACE	Commitment to the Workplace	2	CW	_
WORK-INDIVIDUAL INTERFACE	Work Engagement	3	WE	3
WORK-INDIVIDUAL INTERFACE	Work Life Conflict	7	WF	7
WORK-INDIVIDUAL INTERFACE	Insecurity over working conditions	3	IW	3
WORK-INDIVIDUAL INTERFACE	Intention to Leave	2	ITL	2
WORK-INDIVIDUAL INTERFACE	Job Insecurity	3	JI	3
WORK-INDIVIDUAL INTERFACE	Job Satisfaction	7	JS	7
WORK ORGANIZATION AND JOB CONTENTS	Control over working Time	2	CT	_
WORK ORGANIZATION AND JOB CONTENTS	Possibilities for Development	2	PD	_
WORK ORGANIZATION AND JOB CONTENTS	Variation of Work	1	VA	_
WORK ORGANIZATION AND JOB CONTENTS	Meaning of Work	2	MW	_
DEMANDS AT WORK	Demands for Hiding Emotions	2	HE	
DEMANDS AT WORK	Emotional Demands	2	ED	2
INTERPERSONAL RELATIONS AND LEADERSHIP	Influence	3	IN	3
INTERPERSONAL RELATIONS AND LEADERSHIP	Predictability	2	PR	_

INTERPERSONAL RELATIONS AND LEADERSHIP	Quality of leadership	4	QL	4
INTERPERSONAL RELATIONS AND LEADERSHIP	Recognition	1	RE	1
INTERPERSONAL RELATIONS AND LEADERSHIP	Role Clarity	1	CL	1
INTERPERSONAL RELATIONS AND LEADERSHIP	Role Conflicts	3	CO	3
INTERPERSONAL RELATIONS AND LEADERSHIP	Sense of Community at Work	2	SW	2
INTERPERSONAL RELATIONS AND LEADERSHIP	Social Support from Colleagues	3	SC	3
INTERPERSONAL RELATIONS AND LEADERSHIP	Social Support from Supervisor	4	SS	4
CONFLICTS AND OFFENSIVE BEHAVIORS	Mobbing	5	MO	_
CONFLICTS AND OFFENSIVE BEHAVIORS	Physical violence	1	PV	_
CONFLICTS AND OFFENSIVE BEHAVIORS	Threats of Violence	1	TV	_
CONFLICTS AND OFFENSIVE BEHAVIORS	Unpleasant Teasing	1	UT	_
SOCIAL CAPITAL	Organizational Justice	2	JU	2
SOCIAL CAPITAL	Vertical Trust	2	TM	2
PHYSICAL WORK ENVIRONMENT	Physical Work Environment	5	PWE	_
DEMANDS AT WORK	Quantitative Demands	3	QD	3
DEMANDS AT WORK	Work Pace	2	WP	2
TOTAL ITEMS		108		79

Table S2: Occupation Categories and their ISCO-08 mappings

**Occupation Name**	ISCO-08 codes
Admin, leading	11, 12
Tech, leading	13, 14
Workers and Other	92
Tech and Engineering: engineers	21
Tech: technicians	25
Admin and Educational Services, not leading	23, 24
Tech Services, not leading	31
Other prof. (health-safety services & other professionals)	21, 22, 32

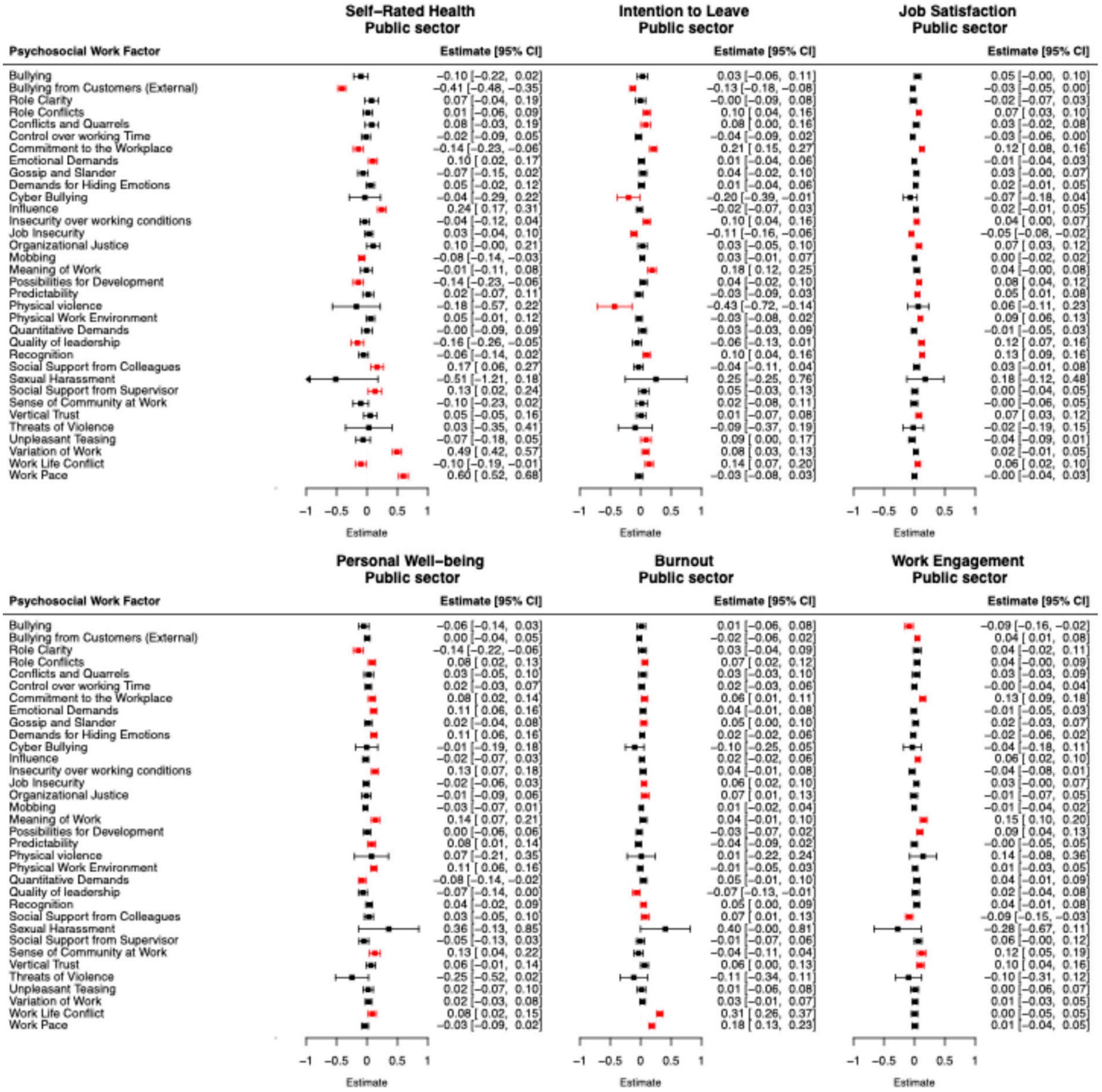

Figure S1A: Estimated coefficients and 95% confidence intervals for the psychosocial work factors regressed with self-rated health, intention to leave, job satisfaction, personal wellbeing, burnout and work engagement in a subgroup analysis for the **Public sector**. Red color indicates estimates with p-value less than 0.05.

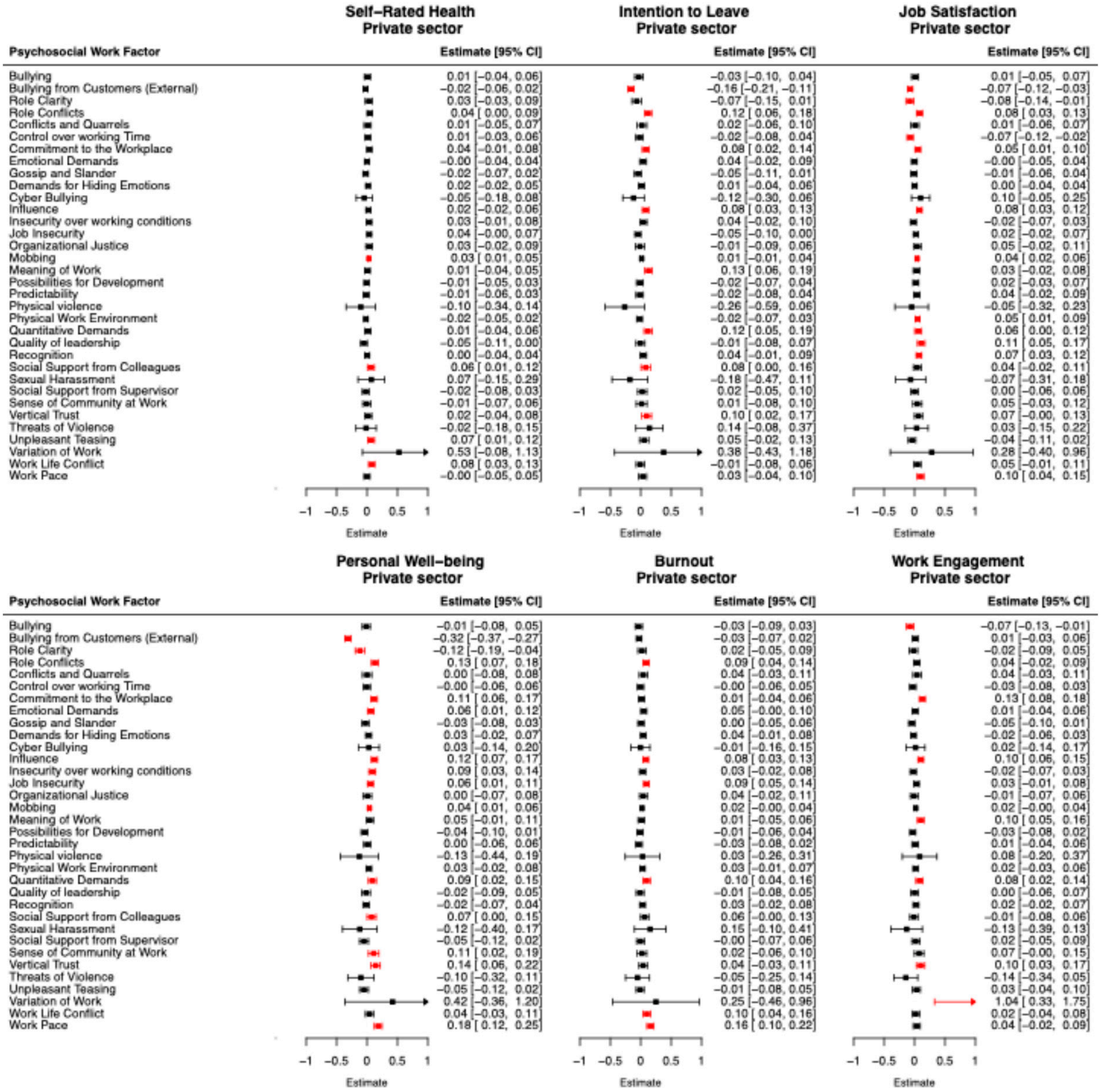

Figure S1B: Estimated coefficients and 95% confidence intervals for the psychosocial work factors regressed with self-rated health, intention to leave, job satisfaction, personal wellbeing, burnout and work engagement in a subgroup analysis for the **Private sector**. Red color indicates estimates with p-value less than 0.05.

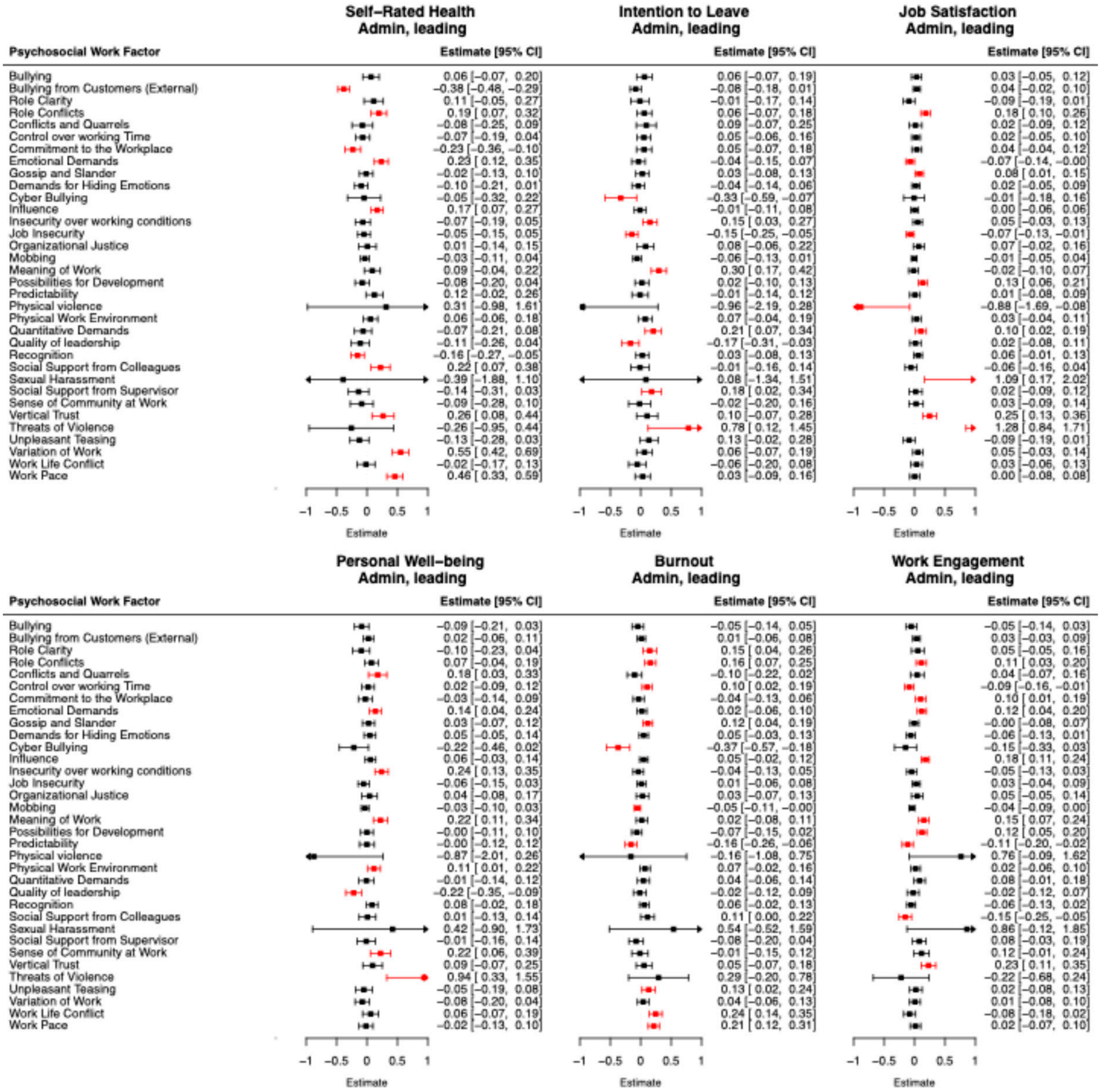
Bullying
Bullying from Customers (External)
Role Clarity
Role Conflicts
Conflicts and Quarrels
Control over working Time
Commitment to the Workplace
Emotional Demands
Gossip and Slander
Demands for Hiding Emotions
Cyber Bullying
Influence
Insecurity over working conditions
Job Insecurity
Organizational Justice
Mobbing
Meaning of Work
Possibilities for Development
Predictability
Physical violence
Physical Work Environment
Quantitative Demands
Quality of leadership
Recognition
Social Support from Colleagues
Sexual Harassment
Social Support from Supervisor
Sense of Community at Work
Vertical Trust
Threats of Violence
Unpleasant Teasing
Variation of Work
Work Life Conflict
Work Pace

Figure S2A: Estimated coefficients and 95% confidence intervals for the psychosocial work factors regressed with self-rated health, intention to leave, job satisfaction, personal wellbeing, burnout and work engagement in a subgroup analysis for **Admin, leading**. Red color indicates estimates with p-value less than 0.05.

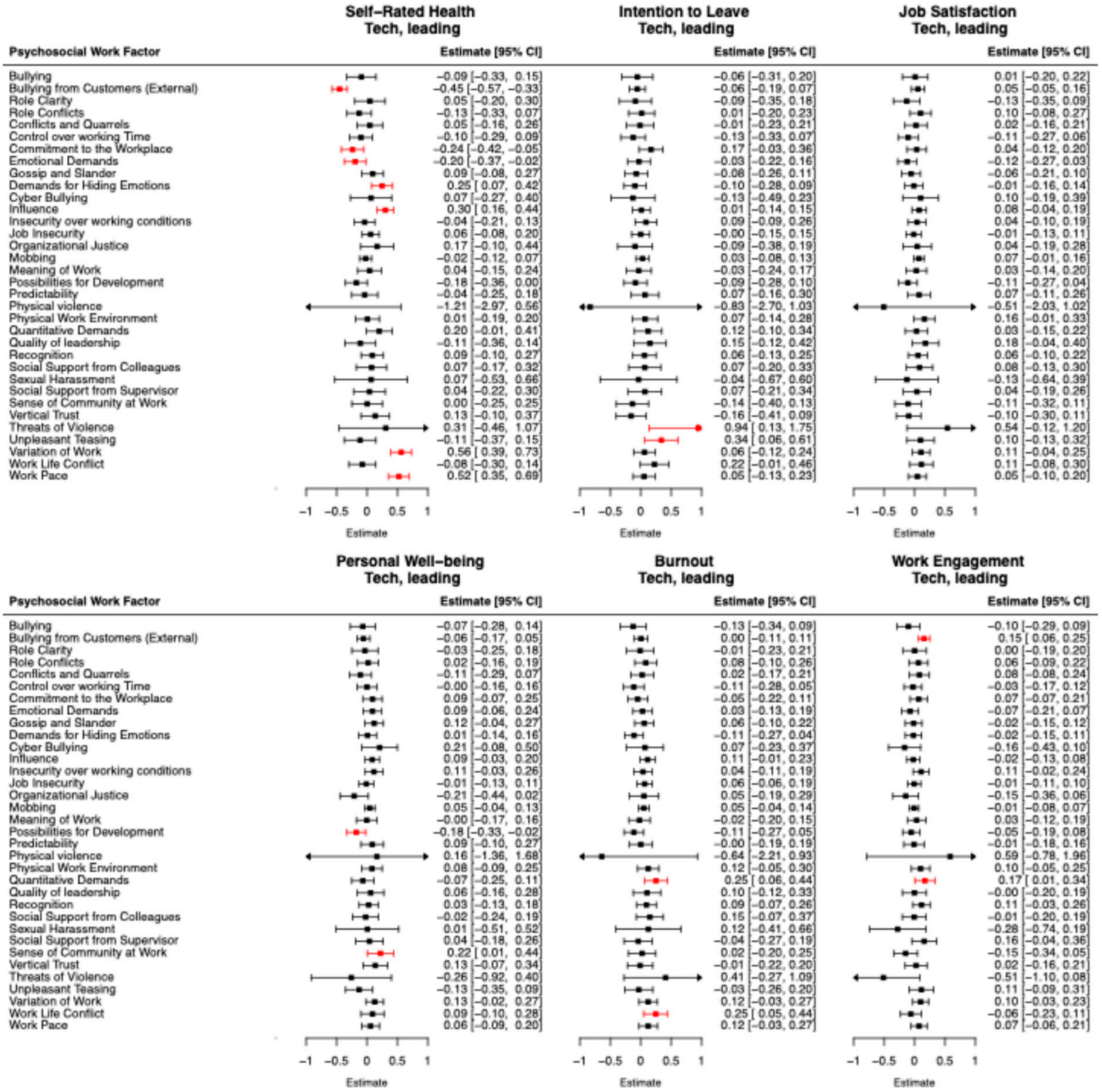

Figure S2B: Estimated coefficients and 95% confidence intervals for the psychosocial work factors regressed with self-rated health, intention to leave, job satisfaction, personal wellbeing, burnout and work engagement in a subgroup analysis for **Tech, leading**. Red color indicates estimates with p-value less than 0.05.

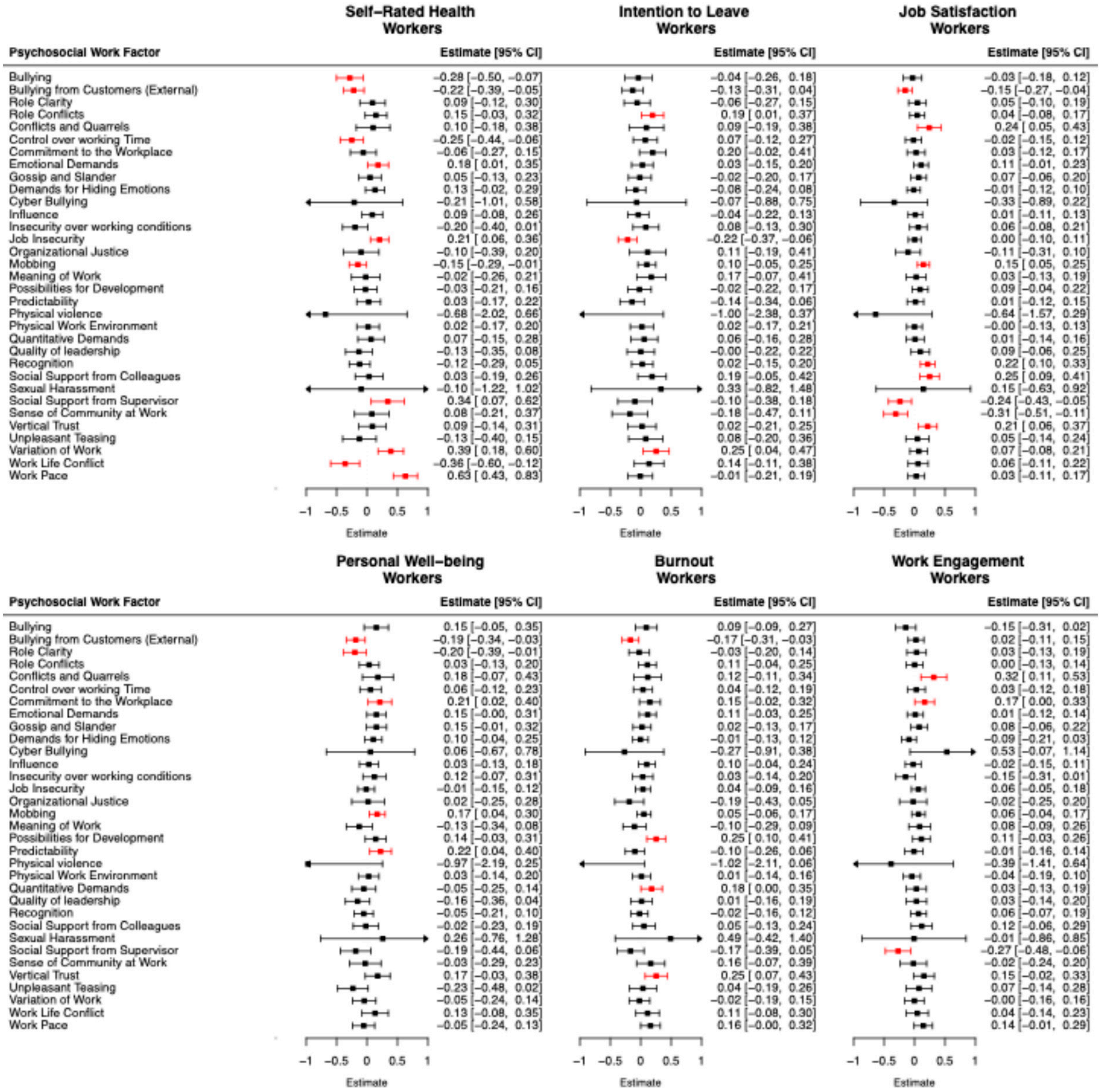

Figure S2C: Estimated coefficients and 95% confidence intervals for the psychosocial work factors regressed with self-rated health, intention to leave, job satisfaction, personal wellbeing, burnout and work engagement in a subgroup analysis for **Workers**. Red color indicates estimates with p-value less than 0.05. Note that Threat of Violence is linearly dependent with Physical Violence in this subgroup and therefore its coefficient is not estimated.

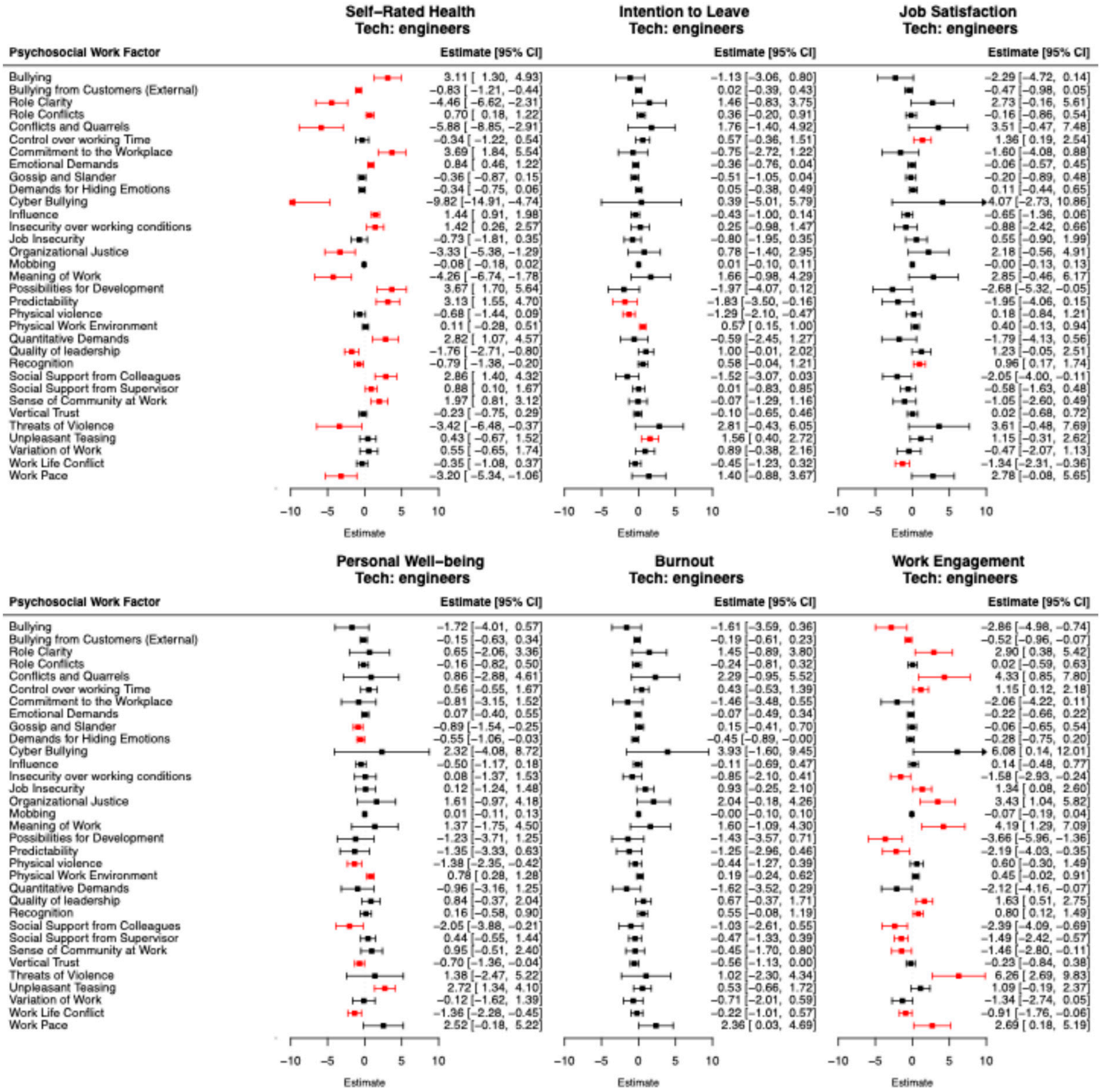

Figure S2D: Estimated coefficients and 95% confidence intervals for the psychosocial work factors regressed with self-rated health, intention to leave, job satisfaction, personal wellbeing, burnout and work engagement in a subgroup analysis for **Tech: engineers**. Red color indicates estimates with p-value less than 0.05. Note that Sexual Harassment is 0 for all participants in this subgroup and therefore its coefficient is not estimated.

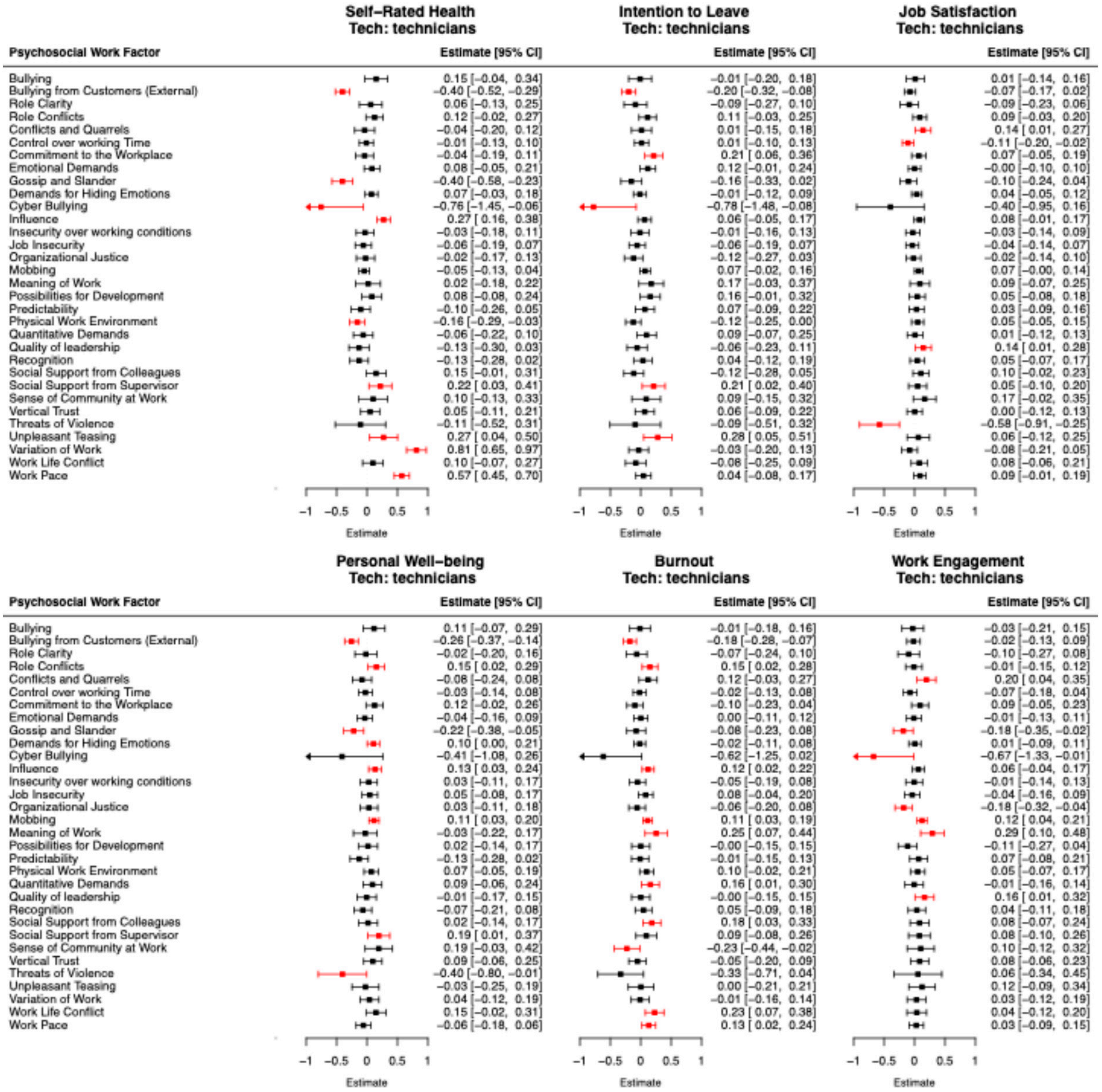

Figure S2E: Estimated coefficients and 95% confidence intervals for the psychosocial work factors regressed with self-rated health, intention to leave, job satisfaction, personal wellbeing, burnout and work engagement in a subgroup analysis for **Tech: technicians**. Red color indicates estimates with p-value less than 0.05. Note that Sexual Harassment and Physical Violence are 0 for all participants in this subgroup and therefore their coefficients are not estimated.

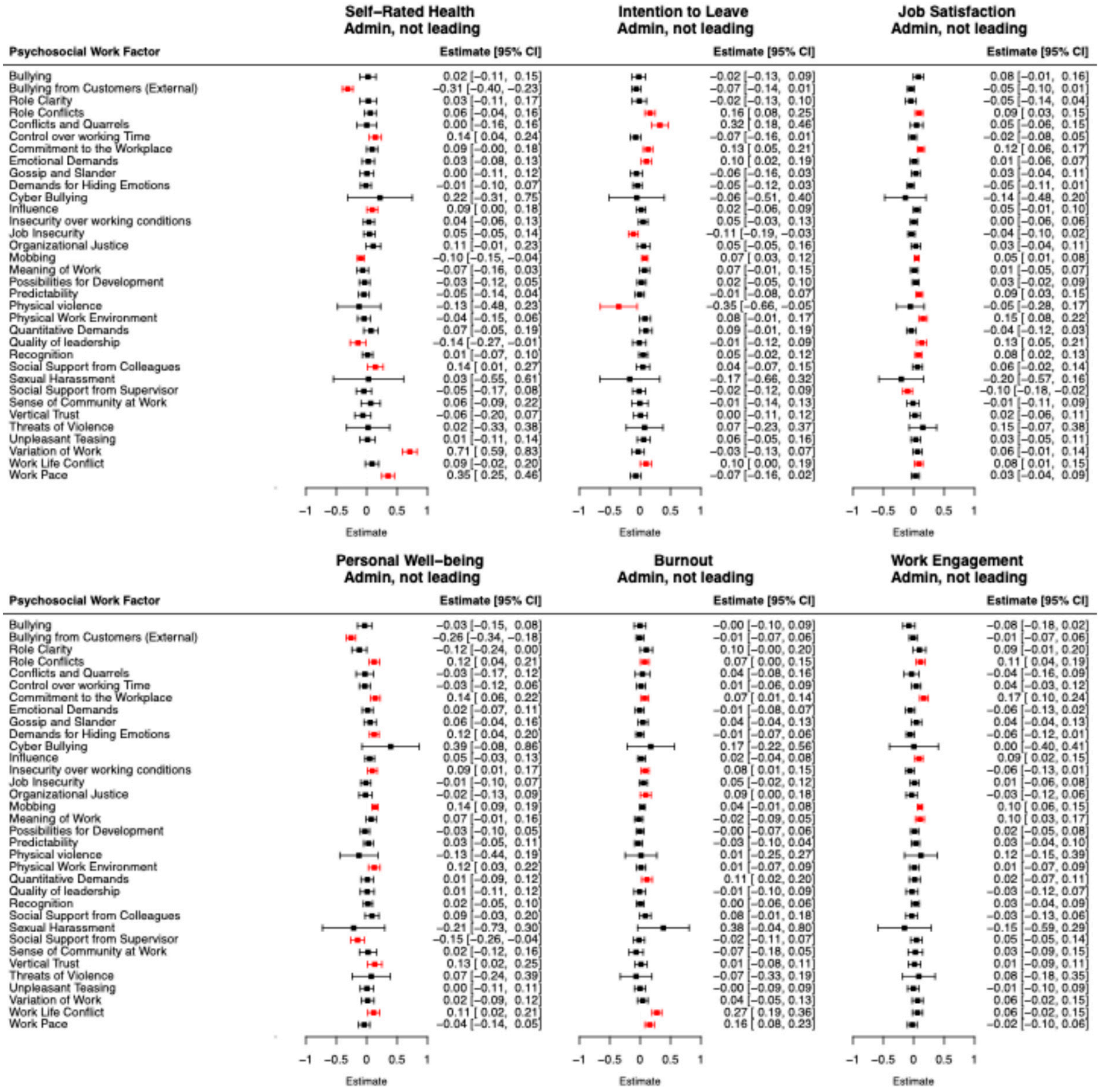

Figure S2F: Estimated coefficients and 95% confidence intervals for the psychosocial work factors regressed with self-rated health, intention to leave, job satisfaction, personal wellbeing, burnout and work engagement in a subgroup analysis for **Admin, not leading**. Red color indicates estimates with p-value less than 0.05.

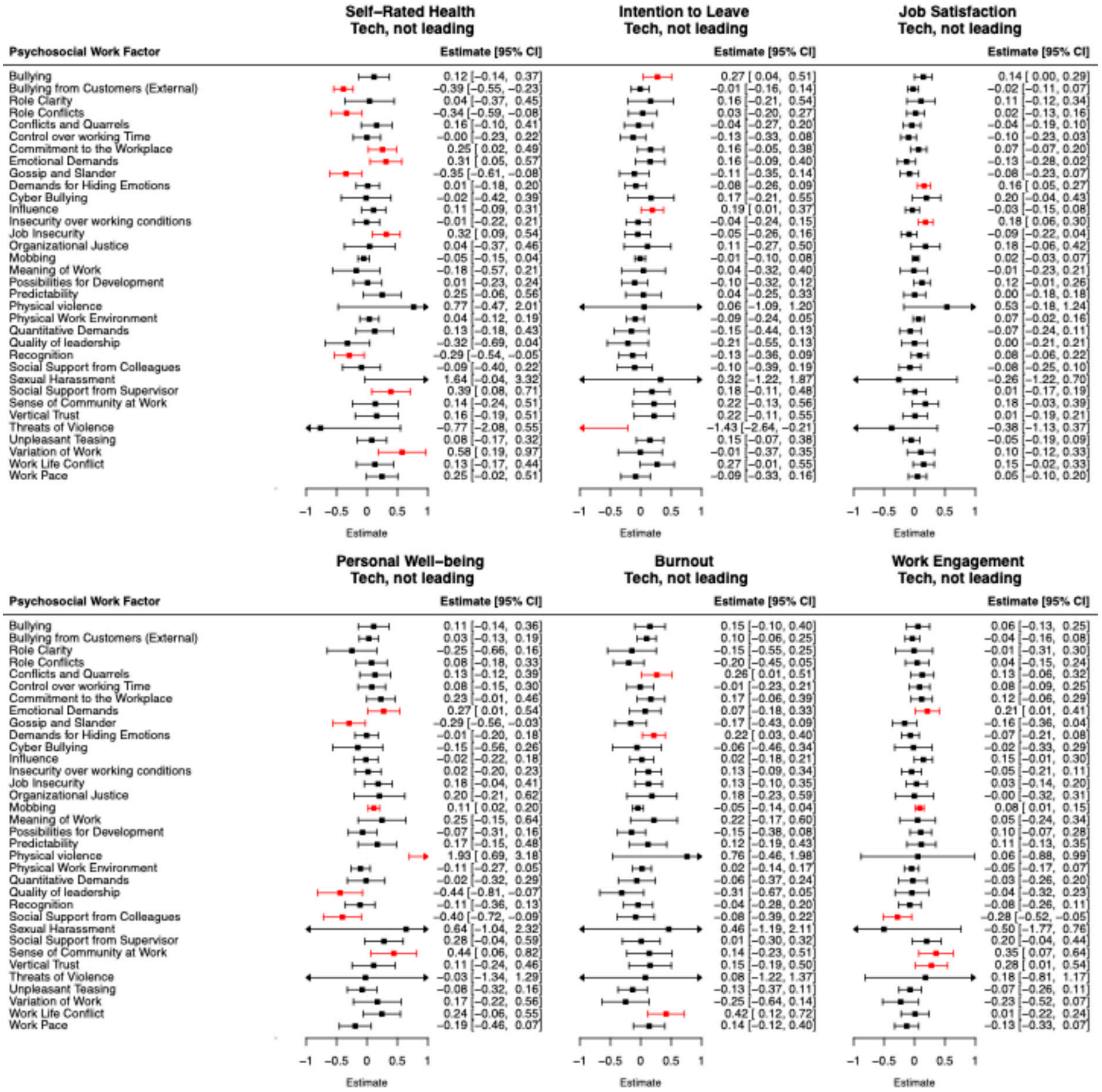

Figure S2G: Estimated coefficients and 95% confidence intervals for the psychosocial work factors regressed with self-rated health, intention to leave, job satisfaction, personal wellbeing, burnout and work engagement in a subgroup analysis for **Tech, not leading**. Red color indicates estimates with p-value less than 0.05.

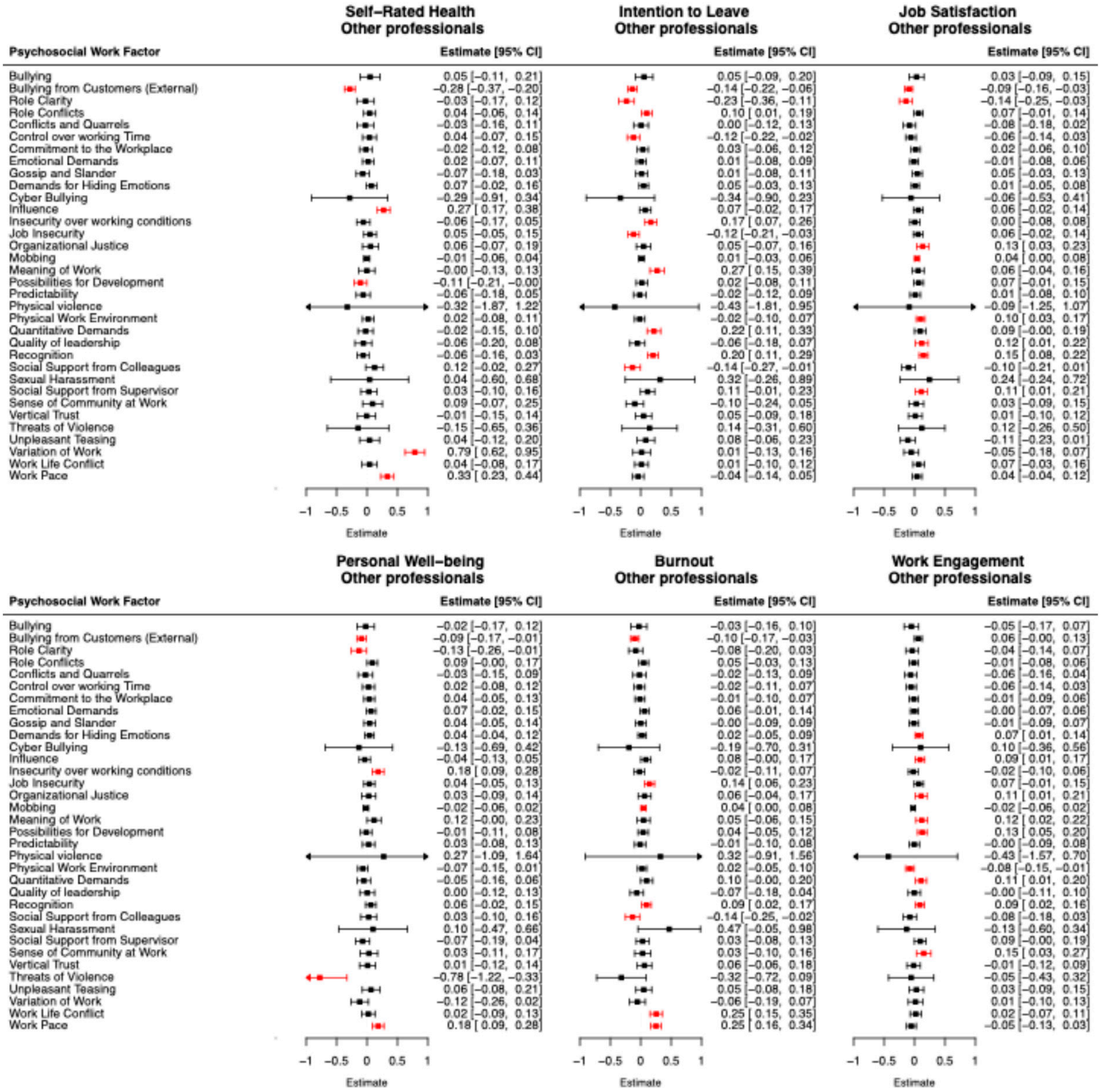

Figure S2H: Estimated coefficients and 95% confidence intervals for the psychosocial work factors regressed with self-rated health, intention to leave, job satisfaction, personal wellbeing, burnout and work engagement in a subgroup analysis for **Other professionals**. Red color indicates estimates with p-value less than 0.05.
